# Development of Tinnitus at a Low Dose of Sertraline: Clinical Course and Proposed Mechanisms

**DOI:** 10.1155/2016/1790692

**Published:** 2016-09-15

**Authors:** Christopher W. T. Miller

**Affiliations:** University of Maryland School of Medicine, Baltimore, MD, USA

## Abstract

*Introduction*. Serotonin is involved in filtering of auditory stimuli. Cochlear input is processed through complex interactions between serotonergic, glutamatergic, and GABAergic neurotransmitter systems. Options for treatment of tinnitus include selective serotonin reuptake inhibitors (SSRIs); however in rare instances this symptom may occur as a side effect of this class of medications.* Case Presentation*. A 50-year-old woman developed bilateral tinnitus after several weeks of being treated with sertraline 50 mg. She had been on a long-standing daily dose of aspirin 325 mg which had been discontinued shortly before starting sertraline. Medical work-up was negative for her symptom. Shortly after discontinuation of the medication, her tinnitus subsided completely.* Discussion*. Tinnitus is a rare side effect of sertraline and may be related to particular distribution of serotonin receptor subtypes within the auditory system, and serotonergic agents may reinforce or desensitize the activity of different receptors. Also, there may be a priming effect of salicylate agents on the auditory system, predisposing particular patients to be more sensitive to how auditory stimuli are processed.

## 1. Introduction

Serotonin has a role in the filtering of auditory input [1], allowing for appropriate disregarding of trivial sensory input [2], and the development of tinnitus may reflect a disturbance in this function [2]. Serotonergic receptors have been described in the cochlea, originating mainly from the dorsal region of the superior olivary complex [3], and this neurotransmitter seems to have a close correlation with GABAergic and glutamatergic input into the cochlea. In effect, serotonin can hamper the glutamatergic bursts which are observed in cochlear pathology [4]. In terms of the subtypes of serotonin receptors distributed in the cochlea [5], some may have a direct influence on glutamatergic functioning, such as the postsynaptic 5-HT1A receptor, which has a role in inhibiting adenylyl cyclase.

While tinnitus can be associated with a withdrawal period from selective serotonin reuptake inhibitors (SSRIs) and these medications (including sertraline) have actually been utilized to treat tinnitus [6] (through enhancement of inhibitory GABAergic interneurons), there is still conflicting evidence on how exactly the serotonergic drugs influence perception and processing of auditory stimuli [7]. This writer reports the case of a patient who developed relatively rapid-onset tinnitus while on a rather low dose of sertraline.

## 2. Case Presentation

A 50-year-old Caucasian woman presented for management of stress and anxiety. She worked in a busy office and was noticing the pressures of her job were having an effect on her mood, which had become more dysphoric over time; she also described “panic-type” feelings when she was feeling overwhelmed. She described her sleep as fragmented. There were no safety concerns. There was no clear decrease in functionality. She did not have any significant past psychiatric or medical history. She was not actively taking any psychiatric medications. She had been taking aspirin 325 mg once daily for several years (self-initiated and maintained) but had stopped before coming to the clinic (there was no stated reason for discontinuation). There was no significant substance history. Working diagnoses of unspecified depressive disorder and unspecified anxiety disorder were established; structured rating scales were not utilized. After discussing our treatment options, as well as their risks and benefits, sertraline 50 mg once daily was started.

The patient reported a positive effect on her mood and anxiety symptoms and reported a near-complete improvement in her anxiety. Approximately five weeks after being treated with this regimen, the patient began to experience bilateral, high-pitched tinnitus, something she had never experienced before. There had been no new additions to her medication regimen or other appreciable changes in well-being. Assessments by her primary care physician and an otolaryngologist yielded negative results. It was not felt that the long-standing treatment with aspirin (which had ended recently) was the culprit for the symptom, as the tinnitus had never been present before. The patient was able to continue working but mentioned that the tinnitus was having a serious impact on her ability to function and enjoy life.

Upon further discussion, it was decided to try to discontinue sertraline. At this point, the patient had been on the medication for around nine weeks. Within a few days of stopping the medication, she noticed the tinnitus beginning to subside, and after around one week it was completely resolved.

## 3. Discussion

Sertraline has generally not been associated with the development of tinnitus [8], though it has been described during the period of medication withdrawal [9]. A single earlier case report described the development of tinnitus in a patient after sertraline administration. In this case, tinnitus failed to remit after discontinuation but was eventually resolved with the use of mirtazapine [10], suggesting that perhaps this antidepressant or one with a similar effect on serotonin subtypes may be considered in cases of tinnitus (i.e., inhibiting 5-HT2A and 5-HT2C). However, given that sertraline has been used successfully as a therapeutic agent for tinnitus [6], the relationship is far from straightforward and the effect is difficult to predict.

In addition to the direct interplay serotonin has with the GABA and glutamate systems, something potentially relevant to this case is that salicylates can activate serotonergic neurons in the dorsal raphe through relays from the dorsal cochlear nucleus [11], thus potentially priming the neurons for an enhanced perception of tinnitus, due to hyperactivity in the dorsal cochlear nucleus, inferior colliculus, and auditory cortex [11, 12].

The distribution of key serotonin receptors has been studied in auditory signal processing. There does seem to be representation of 5-HT1A receptors in the organ of Corti and spiral ganglion [5], as well as 5-HT2A receptors in the auditory cortex [13]. As opposed to the more reliably inhibitory effect of 5-HT1A, the effects of 5-HT2A agonism in cortical areas involved in processing auditory signals may evoke neuronal excitation or inhibition [14, 15], as there is a close correlation with GABAergic interneurons as well as the glutamatergic system [16]. Thus, an overly primed serotonergic pathway may lead to unpredictable sensory filtration of auditory stimuli, which may as a result be enhanced. In addition, 5-HT2C (present on the spiral ganglion [5]) has also been shown to participate in neuronal network excitability, and polymorphism of this receptor has been shown to increase sensitivity to auditory stimuli [2]. In rodents, selective serotonin reuptake inhibitors have been shown to desensitize this receptor over time [17], an effect which may underlie some of the subsequent activating effects through release of norepinephrine and dopamine, but which also has relevance in processing of auditory stimuli.

In conclusion, this case illustrates that there is a finely tuned serotonergic influence on the processing of auditory signals, and there may be enhanced sensitivity and perception to certain stimuli. This writer hypothesizes as well that a priming effect in this patient may have led to disruption of normal hearing and heightened her perception, leading to tinnitus.
